# *Cladophora glomerata* enriched by biosorption with *Mn(II)* ions alleviates lipopolysaccharide‐induced osteomyelitis‐like model in MC3T3‐E1, and 4B12 osteoclastogenesis

**DOI:** 10.1111/jcmm.15294

**Published:** 2020-06-04

**Authors:** Lynda Bourebaba, Izabela Michalak, Meriem Baouche, Katarzyna Kucharczyk, Andrzej M. Fal, Krzysztof Marycz

**Affiliations:** ^1^ Department of Experimental Biology Faculty of Biology and Animal Science Wrocław University of Environmental and Life Sciences Wrocław Poland; ^2^ International Institute of Translational Medicine Wisznia Mała Poland; ^3^ Department of Advanced Material Technologies Faculty of Chemistry Wrocław University of Science and Technology Wrocław Poland; ^4^ Collegium Medicum Institute of Medical Science Cardinal Stefan Wyszyński University (UKSW) Warsaw Poland

**Keywords:** biosorption, *Cladophora glomerata*, LPS, manganese, osteoclast, osteomyelitis

## Abstract

Chronic osteomyelitis, a bone infectious disease, is characterized by dysregulation of bone homeostasis, which results in excessive bone resorption. Lipopolysaccharide (LPS) which is a gram‐negative endotoxin was shown to inhibit osteoblast differentiation and to induce apoptosis and osteoclasts formation in vitro. While effective therapy against bacteria‐induced bone destruction is quite limited, the investigation of potential drugs that restore down‐regulated osteoblast function remains a major goal in the prevention of bone destruction in infective bone diseases. This investigation aimed to rescue LPS‐induced MC3T3‐E1 pre‐osteoblastic cell line using the methanolic extract of *Cladophora glomerata* enriched with *Mn(II)* ions by biosorption. LPS‐induced MC3T3‐E1 cultures supplemented with *C. glomerata* methanolic extract were tested for expression of the main genes and microRNAs involved in the osteogenesis pathway using RT‐PCR. Moreover, osteoclastogenesis of 4B12 cells was also investigated by tartrate‐resistant acid phosphatase (TRAP) assay. Treatment with algal extract significantly restored LPS‐suppressed bone mineralization and the mRNA expression levels of osteoblast‐specific genes such as runt‐related transcription factor 2 (*Runx2*), alkaline phosphatase (*ALP*) and osteocalcin (*OCN*), osteopontin (*OPN*), *miR‐27a* and *miR‐29b*. The extract also inhibited osteoblast apoptosis, significantly restored the down‐regulated expression of *Bcl‐2*, and decreased the loss of MMP and reactive oxygen spices (ROS) production in MC3T3‐E1 cells induced by LPS. Furthermore, pre‐treatment with algal extract strongly decreased the activation of osteoclast in MC3T3‐E1‐4B12 coculture system stimulated by LPS. Our findings suggest that *C. glomerata* enriched with *Mn(II)* ions may be a potential raw material for the development of drug for preventing abnormal bone loss induced by LPS in bacteria‐induced bone osteomyelitis.

## 1 INTRODUCTION

Bone is considered as being a highly dynamic tissue undergoing continuous remodelling cycles, consisting of bone neoformation trough osteoblast differentiation and osteoclast‐induced resorption. Bone remodelling is a fundamental process for the reconstruction of fractured bones, adaptation of the skeleton to mechanical solicitations and calcium homeostasis. Bone replacement and neoformation basically respond to complex mechanisms that can be grouped into three main stages, with bone resorption initiation by osteoclasts, bone resorption transitioning to proper bone formation, and finally bone formation after osteoblasts differentiation. Excessive pathological bone loss often occurs when an imbalance between formation and bone resorption appears, leading to the development of many severe inflammatory conditions such as osteomyelitis, bacterial arthritis and infected orthopaedic implants.^1^, ^2^

Musculoskeletal infections, such as osteomyelitis, are among the most common and most challenging degenerative inflammatory diseases in clinical medicine. These are generally defined as an inflammatory process that could be limited to the bone and the joint, or can propagate to the bone marrow, the periosteum and the surrounding soft tissues, arising from the inoculation of one or more infectious organisms, and thus inducing significant destruction and necrosis of the local bone tissue, sequestrum formation and apposition of new bone tissue.^3^, ^4^ Bone infections are often accompanied by the formation of biofilms responsible for pathological bone resorption and reactive bone formation. These biofilms typically comprise a set of various soluble factors such as proteins, lipids, lipopolysaccharides (LPS) and DNA that are produced and secreted by the bacteria in order to form a protective matrix that will counter antibiotic and immune treatments, thus making the therapeutic management rather hazardous.^5^ LPS, an endotoxin found in the outer membrane of all gram‐negative bacteria, was one of the first bacterial components to be widely implicated in the process of bone resorption observed during infection.^6^ LPS is known to trigger the release of many pro‐inflammatory cytokines from neutrophils and macrophages such as interleukin‐1α (*IL‐1α*) and tumour necrosis factor‐α (*TNF‐α*), which subsequently initiate the inflammatory cascades that causes tissue damage and destruction. It has been proposed that released LPS from infected root canals could modulate the secretion of *IL‐1α* and *TNF‐α* from macrophages, and thus stimulate metalloproteinase‐1 (*MMP‐*1) synthesis that will in turn initiate dramatic bone resorption.^7^ In addition, LPS application to bone marrow cell cultures has been shown to result in the generation of osteoclasts; furthermore, local injection of LPS in the femur resulted in a rapid and massive formation of osteoclasts as well as the expansion of eroded bone areas. Osteoclast formation is a key step in the bone resorption process, as only osteoclasts have the ability to actively resorb while having a limited life span of two weeks at most.^8^ The differentiation of osteoclasts initiated from a cluster of hematopoietic monocyte/macrophage‐like stem cells responds to several systemic and local stimuli, such as hormones, cytokines, growth factors and eicosanoids.^9^ The differentiation of osteoclasts takes place in several stages and is essentially characterized by the emergence of tartrate‐resistant acid phosphatase‐positive cells (*TRAP*), fusion into multinucleate cells, initiation of bone resorption and induction of spontaneous apoptosis.^10^ The cytokine *RANKL* is an essential factor in the initiation and formation of osteoclasts.^11^ Binding of *RANKL* to its receptor triggers the recruitment of the various adaptation factors associated with the TNF receptor (*TRAF*) and activates subsequently several downstream signal pathways, in particular those involving *MAPKs*, *NF‐κBs* and *PI3*K/*Akt*. Then, activation of transcription factors such as activator protein 1 (*AP‐1*) and activated T‐cell cytoplasmic nuclear factor 1 (Nuclear Factor Of Activated T Cells 1) occurs. The recruitment of these downstream factors initiates the differentiation and functioning of osteoclasts by inducing the expression of specific genes, including *TRAP*, cathepsin K (*CTSK*) and matrix metalloproteinase 9 (*MMP‐9*), thus resulting in the final formation of mature osteoclasts.^12^, ^13^

Manganese (Mn), an essential ubiquitous trace element, is known to be required for normal growth, development and cellular homeostasis, and is thus one of the most important minerals to bone tissue. Physiologically, Mn is involved in protein metabolism and regeneration of the connective tissue. Moreover, it is closely associated with certain enzymatic activities such as superoxide dismutase (SOD) and Arginase, as well as metallothionein. Its main function is to activate various enzymes that control the metabolism of carbohydrates, proteins and lipids (including cholesterol) and nitrogen metabolism even in maintaining the normalization of the synthesis and secretion of insulin as well.^14^, ^15^ In the skeleton, manganese positively modulates *RANKL/OPG* ratio during bone formation, determining thus thickness of trabecular bone area and increasing trabecular number.^16^ It has been reported that manganese deficiencies are at the origin of various bone malformations, stunted growth and impaired motor coordination, leading in the long term to osteoporosis development and to the occurrence of congenital disorders of the skeletal system, such as chondrodystrophy; the use of trace element supplementation such as manganese seems to be a plausible strategy for the management of bone homeostasis disorders.^17^ The phyla of macroalgae have been widely recognized to bring a novelty and a diversity of chemical and pharmacological functional ingredients. In addition, algae are considered as real rich sources of various highly bioactive compounds found in marine resources such as polyphenols, pigments, vitamins, carbohydrates, proteins, lipids and minerals.^18^ As one of the most common and important filamentous green algae in freshwater, *Cladophora glomerata* or commonly known as ‘cotton‐mat’ or ‘blanket weed’ is attracting more and more attention not only because of its high nutritional value, but also because of its richness in various secondary metabolites giving it a high potential in therapeutics for the treatment of among others inflammation, oxidative stress and infectious diseases.^19^, ^20^ Recently, the concept of mutual potentiation of the therapeutic and physiological effects of natural substrates and trace elements has been introduced and is part of the new innovative perspectives in therapeutics. Thus, biosorption process can be applied in order to improve the bioavailability of the microelements for the animals while combining the nutritional and curative properties of the used biomasses.^21^, ^22^ In that context, the methanolic extract obtained from *C. glomerata* enriched with *Mn(II)* ions via biosorption process was applied to a model of LPS‐induced osteomyelitis on MC3T3 pre‐osteoblasts cell line, as well as on the osteoclastogenesis process induced on 4B12 cells in the present investigation, with the goal of combining the beneficial effects of manganese on bone metabolism, and the anti‐inflammatory, antiapoptotic and antioxidant effects of *C. glomerata* extract.

## 2 MATERIALS AND METHODS

### 2.1 Chemicals

All chemicals and reagents were obtained from Sigma‐Aldrich; cell culture reagents were purchased from Gibco BRL unless otherwise stated.

### 2.2 Algal biomass

The biomass of freshwater macroalgae—*C. glomerata*, was collected from the surface of the pond in Tomaszówek, Łódź Province, Poland (51°27′21″N, 20°07′43″E) in October 2016. The study was carried out on private land, and the owner of the land gave permission to conduct the study on this site. No specific permissions were required for this location/activity. These studies did not involve endangered or protected species. Then, the biomass was air‐dried and fine milled using grinding mills (Retsch GM 300).

### 2.3 Biosorption process of *Mn(II)* ions by algal biomass

The biosorption of *Mn(II)* ions was carried out according to the procedure described by Michalak and Chojnacka.^23^ In brief, the experiments were performed in Erlenmeyer flasks containing 500 mL of *Mn(II)* ions solution in a shaker at 200 rpm (IKA KS 260 basic; IKA^®^ Works, Inc). The biomass content in the solution was 5 g/L (*C*_S_). The solution of *Mn(II)* ions with the initial concentration of 300 mg/L (*C*_0_) was prepared in deionized water by dissolving appropriate amount of the MnSO_4_·H_2_O salts (from Avantor Performance Materials Poland SA). The initial pH of the solution was adjusted to 5, according to our previous studies,^23^ with 0.1 M NaOH/HCl (from Avantor Performance Materials Poland SA) using pH‐meter Mettler Toledo (Seven Multi) equipped with an electrode InLab413 with compensation of temperature. The biosorption time was 4 hours. After the process, the solution was filtered through filter paper (Macherey‐Nagel, Mn 615). Manganese solution before and after biosorption process was analysed using ICP–OES method (inductively coupled plasma‐optical emission spectrometry) using Varian VISTA MPX spectrometer in the laboratory of Multielemental Analyses at Wrocław University of Science and Technology, which is accredited by ILAC‐MRA and Polish Centre for Accreditation (no. AB 696). The enriched with *Mn(II)* ions biomass was subjected to the extraction process.

### 2.4 Extraction of the enriched with *Mn(II)* ions algal biomass

The enriched with *Mn(II)* ions biomass (5 g) was extracted with 250 mL of methanol (from Avantor Performance Materials Poland SA) by shaking in a shaker at 150 rpm (IKA KS 260 basic) for 48 hours in the darkness.^24^ After filtration through filter paper, the solvent was evaporated on an evaporator (BUCHI Labortechnik AG, Rotavapor R‐100). The remaining dry residue was then dissolved in 50 mL of methanol. The obtained extract was analysed in terms of multielemental analysis using ICP‐OES technique.

### 2.5 Characteristics of algal biomass

*Cladophora glomerata* used in the present study was previously characterized in terms of the content of fatty acids, free and protein‐bound amino acids, total phenols content, vitamin C, E,^25^ as well as the multielemental composition.^26^ This macroalgae is a valuable raw material for many applications.

### 2.6 Cell culture

Murine cell line, MC3T3‐E1 Subclone 4 (ATCC^®^ CRL‐2593™), which is an osteoblast‐like cell line obtained from the C57BL/6 mouse calvaria was purchased from the American Type Culture Collection; pre‐osteoclast 4B12 cell line was kindly provided by the Professor Shigeru Amano from the Division of Microbiology and Immunology, Department of Oral Biology and Tissue Engineering, Meikai University School of Dentistry, Keyakidai, Sakado, Japan. Cells were cultured at 37°C in a 5% CO_2_ atmosphere using α‐modified minimal essential medium (α‐MEM; Gibco BRL) without ascorbic acid and supplemented with 10% heat‐inactivated foetal bovine serum (FBS). Cells were subcultured when they reached 70%‐80% confluence, every 3 days using Trypsin/EDTA solution. All the experiments were done in triplicates with cells of passage 8‐12, at 37°C, under 5% CO_2_ and in humidified conditions.

### 2.7 Cell metabolic activity

Cell metabolic activity was monitored using the Resazurin‐based assay kit (Alamar Blue; Sigma‐Aldrich). Briefly, MC3T3‐E1 cells were plated in 96‐well culture plates at 5 × 10^3^ cells/well and cultured in 100 µL of alpha‐MEM medium. To evaluate the effect of *C. glomerata* extract enriched with *Mn(II)* ions (*CgMn*), as well as *Mn(II)* ions itself on pre‐osteoblasts viability, cells were treated with 0.5%, 1%, 2%, 4% and 8% of *CgMn* or *Mn(II)* for 72 hours. To evaluate the effect of *CgMn* on LPS‐induced pre‐osteoblasts viability, cells were pre‐treated with 0.5% and 1% of *CgMn* for 24 hours and then challenged with 1 µg/mL LPS for additional 24 hours in the absence of FBS. At the end of each related treatment, all media were removed and 100 µL of a 10% resazurin solution was added to each well. Cells were cultured for an additional 2 hours. Afterwards, absorbance was measured at the specific wavelengths: 600 nm for resazurin and 690 nm as a background absorbance using a microplate reader (Spectrostar Nano; BMG Labtech). The effect of the methanolic extract obtained from *C. glomerata* enriched with *Mn(II)* ions via biosorption process on metabolic activity of cells was expressed as mean of metabolized resazurin compared with that of blank that consists of resazurin with medium only.

### 2.8 Evaluation of cell morphology

Changes in cellular morphology were evaluated using confocal microscopy (Observer Z1 Confocal Spinning Disc V.2 Zeiss) with live imaging chamber. Briefly, cells were first treated with the two *C. glomerata Mn(II)*‐enriched extract during 24 hours and then stimulated by LPS for another 24 hours; afterwards, tested cultures were fixed with 4% paraformaldehyde (PFA) at room temperature for 45 minutes. Samples were after that rinsed with Hank's balanced saline solution (HBSS); then, cell membranes were permeabilized using 0.1% Triton X‐100 solution for 15 minutes at room temperature. Actin filaments were stained using atto‐590‐labelled phalloidin at dilution 1:800 with HBSS for 40 minutes, in the dark at room temperature. Nuclei were imaged by mean of diamidino‐2‐phenylindole (DAPI), using the ProLong™ Diamond Antifade Mountant with DAPI (Invitrogen™). Mitochondria were labelled using the Mito Red fluorescence dye diluted at 1:1000 in culture medium and incubated for 30 minutes at 37°C in a CO_2_ incubator, prior to PFA fixation. Confocal microscope images were acquired as z‐stacks having a z‐interval of 15, 20 or 25 µm between two consecutive optical slices at a digital size of 512 × 512 pixels, and captured with a Canon PowerShot camera. Obtained photomicrographs were merged and analysed using ImageJ software.

### 2.9 Detection of apoptosis by Annexin V labelling

The degree of apoptosis in LPS‐induced MC3T3‐E1 cell population after *C. glomerata* treatment was established using the Muse Annexin V & Dead Cell Assay kit™ (Merck Millipore), based on the detection of phosphatidylserine (PS) on the surface of apoptotic cells according to the manufacturer's protocol. All treated and untreated groups of cells as described above were collected, washed with HBSS and labelled with the Annexin V & Dead Cell Kit for 20 minutes at room temperature. The apoptotic ratio was calculated by identifying four populations: (a) non‐apoptotic cells, not undergoing detectable apoptosis: Annexin V (−) and 7‐AAD (−); (b) early apoptotic cells, Annexin V (+) and 7‐AAD (−); (c) late apoptotic cells, Annexin V (+) and 7‐AAD (+); and (d) cells that have died through non‐apoptotic pathway: Annexin V (−) and 7‐AAD (+), and counted by the use of Muse Cell Analyzer (Merck Millipore).

### 2.10 Analysis of changes in mitochondrial transmembrane potential

Measurement of changes in mitochondria membrane potential (ΔΨm) was performed with the Muse™ MitoPotential Assay kit (Merck Millipore). This flow cytometry‐based assay differentiates four populations of cells: live cells with depolarized mitochondrial membrane; MitoPotential−/7‐AAD−, live cells with intact mitochondrial membrane; MitoPotential+/7‐AAD−, dead cells with depolarized mitochondrial membrane; MitoPotential+/7‐AAD+ and dead cells with intact mitochondrial membrane; and MitoPotential−/7‐AAD+. After LPS challenging of *Mn(II)*‐enriched *C. glomerata* extract‐pre‐treated MC3T3‐E1 cells during 24 hours, cultures were washed with HBSS and stained with the provided fluorescent dyes for 30 minutes at 37°C, and the percentage of total depolarized cells (depolarized live + depolarized dead) was established by the mean of a Muse Cell Analyzer (Merck Millipore).

### 2.11 Quantification of multicaspase activity

Multicaspases activity was quantified by using the Muse MultiCaspase assay kit (Merck Millipore). MC3T3‐E1 cells were pre‐treated with 0.5% and 1% of methanolic extract from *Mn(II)*‐enriched *C. glomerata* for 24 hours and exposed to 1 µg/mL of LPS, and multicaspase activity was subsequently assessed according to the manufacturer's instructions, using the Muse Cell Analyzer (Millipore).

### 2.12 Determination of intracellular reactive oxygen species

Quantitative measurements of intracellular ROS namely Superoxide radicals were established using the flow cytometry‐based analysis by means of Muse^®^ Oxidative Stress Kit (Merck Millipore) according to the users’ guide instructions. Cells were cultured in the presence of methanolic extract from *Mn(II)*‐enriched *C. glomerata* (0.5% and 1%) and LPS during 24 hours for each related treatment; all treated and control untreated cells were subsequently rinsed with HBSS and stained with the related fluorescent dye for 30 minutes at 37°C. Determination of ROS^+^ vs ROS^−^ populations was achieved using a Muse Cell Analyzer (Merck Millipore).

### 2.13 Coculture system of MC3T3‐E1 with 4B12 cells

Transwell cell culture inserts (Greiner Bio‐One) with 3 μm pore‐size filters were placed in individual wells of 24‐well plates. Briefly, 4B12 osteoclastogenic precursor cells (5 × 10^4^ cells/well) were seeded and grown on the well plates; subsequently, pre‐osteoblastic MC3T3‐E1 cells (3 × 10^4^ cells/mL) were seeded and grown on the transwell inserts on top. Pre‐osteoblasts were then supplemented with the two concentrations of extract from *Mn(II)*‐enriched *C glomerate* and challenged with LPS for osteoclastogenic factors production. Cocultures were thus maintained for 10 days in standard culture conditions. On the 11th day, inserts were discarded, and osteoclastogenic‐4B12 differentiated cells were collected for TRAP staining, RT‐PCR analysis and Phalloidin labelling as previously described for confocal evaluation.

### 2.14 Tartrate‐resistant acid phosphatase (TRAP) staining

After 10 days of incubation as described in Section 2.13, 4B12 cells were stained for TRAP using a leukocyte acid phosphatase staining kit (Sigma) according to the manufacturer's instructions. Cells were washed with HBSS and fixed with the provided fixing solution (18 mmol/L citrate solution; acetone and 37% formaldehyde) for 30 seconds and subsequently rinsed thoroughly with deionized water. Later, TRAP staining was performed for 1 hour at 37°C in the dark by immersion of fixed samples in 7 mg/mL Fast Garnet GBC Base solution, 0.1 M sodium nitrite solution, 2.5 mol/L acetate solution, 0.335 mol/L tartrate solution and 12.52 mg/mL naphthol AS‐BI Phosphate solution. After washing‐off the remaining fixing mixture with deionized water, cell nuclei were stained for 2 minutes using haematoxylin solution followed by rinsing in tap water. Stained samples were imaged using an inverted microscope (AxioObserverA1; Zeiss), and pictures were acquired using a Cannon PowerShot digital camera.

### 2.15 Quantification of osteogenic and osteoclastogenic‐related genes expression

Total RNA was isolated from MC3T3‐E1 or 4B12 cells by using Trizol reagent (Sigma) as preconized by the supplier. RNA purity and concentration were established using a nanospectrophotometer (WPA, Biowave II). Genomic DNA (gDNA) digestion and cDNA synthesis were performed by reverse transcription reaction with oligo(dT) primers using a Tetro cDNA Strand cDNA Synthesis Kit (Bioline) by the mean of a T100 Thermal Cycler (Bio‐Rad) according to the provided kit instructions. Real‐time PCR analysis was used to quantify the transcripts of osteogenic and osteoclastogenic‐related genes (Table 1). SensiFAST SYBR Green Kit (Bioline) was used for detection of the target mRNA expressions in a CFX Connect™ Real‐Time PCR Detection System (Bio‐Rad). A total of 150 ng of cDNA were amplified in a total volume of 10 µL containing SYBR Green Master Mix, forward and reverse primers and tested samples. The thermal profile conditions were as follows: 95°C for 2 minutes, followed by 40 cycles at 95°C for 15 seconds, annealing for 15 seconds, and elongation at 72°C for 15 seconds. RT‐qPCR reactions were carried out in triplicate. The relative expression levels of all studied genes were normalized to the expression of house‐keeping gene glyceraldehyde‐3‐phosphate dehydrogenase (GAPDH).

**TABLE 1:** Sequences of primers used in qPCR

Gene	Primer	Sequence 5′‐3′	Amplicon length (bp)	Accession no.
*ALP*	F:	CCTGCTCTGTTTCTTCACCTGT	218	NM_001287172.1
	R:	GGTCTCTCTCTTTCTCTGGCACAAA		
*Runx2*	F:	TCCGAAATGCCTCTGCTGTT	228	NM_001146038.2
	R:	GCCACTTGGGGAGGATTTGT		
*RANKL*	F:	TCCTGTACTTTCGAGCGCAG	192	AF019048.1
	R:	GTTGCAGTTCCTTCTGCACG		
*TRAP*	F:	GTCTCTGGGGGACAATTTCTACT	241	M99054
	R:	GTTTGTACGTGGAATTTTGAAGC		
*c‐Fos*	F:	CCAGTCAAGAGCATCAGCAA	248	BC029814
	R:	TAAGTAGTGCAGCCCGGAGT		
*OCN*	F:	CTCCTGAGAGTCTGACAAAGCCTT	174	NM_007541.3
	R:	GCTGTGACATCCATTACTTGC		
*OPN*	F:	AGACCATGCAGAGAGCGAG	340	NM_001204203.1
	R:	GCCCTTTCCGTTGTTGTCCT		
*NF‐kappa‐B*	F:	CCAACTGGCAGGGGACATGAA	147	L28118.1
	R:	GACTCAGCCGGAAGGCATTG		
Nuclear Factor Of Activated T Cells 1	F:	TTCGAGTTCGATCAGAGCGG	73	NM_016791.4
	R:	AGGTGACACTAGGGGACACA		
*OPG*	F:	TAAGGAACCTCCACGCACAC	100	AY577781.1
	R:	ACTTCCGGATTTGGATGGGG		
*Bcl‐2*	F:	AACATCGCCCTGTGGATGAC	117	NM_009741.5
	R:	AAACAGAGGTCGCATGCTGG		
*Bax*	F:	GATCCAAGACCAGGGTGGC	115	NM_007527.3
	R:	CTTCCAGATGGTGAGCGAGG		
*p21*	F:	ACCGAGCCTGTTTCTCTGTG	113	AF035683.1M
	R:	TCAGGGCTGTACGTGCAAAT		
*p53*	F:	TGAAGACCAAGAAGGGCCAG	106	AB017816.1
	R:	TGGTGATGTGGGACGGGAT		
*Casp‐3*	F:	GAGCTTGGAACGGTACGCTAA	121	NM_001284409.1
	R:	CCAGAGTCCACTGACTTGCT		
*Casp‐9*	F:	ATTCAGCAGGCAGGATCTGG	118	NM_015733.5
	R:	GGCCTGTGTCCTCTAAGCAG		
*GAPDH*	F:	TGCACCACCAACTGCTTAG	177	XM_017321385.1
	R:	GGATGCAGGGATGATGTTC		

Note

Sequences: amplicon length and access numbers of the primer sets*.*

Abbreviations: *ALP*, alkaline phosphatase; *Bax*, BCL‐2‐associated X protein; *Bcl‐2*, B‐cell lymphoma 2; *Casp‐3*, caspase 3; *Casp‐9*, caspase 9; *c‐Fos*, proto‐oncogene for activator protein‐1; *GADPH*, glyceraldehyde‐3‐phosphate dehydrogenase; Nuclear Factor Of Activated T Cells 1, nuclear factor of activated T‐cell, cytoplasmic 1; *NF‐kappa‐B*, nuclear factor kappa‐light‐chain‐enhancer of activated B cells; *OCN*, osteocalcin; *OPG*, osteoprotegerin; *OPN*, osteopontin; *p21*, cyclin‐dependent kinase inhibitor 1A; *p53*, tumour suppressor p53; *RANKL*, receptor activator of nuclear factor kappa‐Β ligand; *Runx2*, runt‐related transcription factor 2; *TRAP*, tartrate‐resistant acid phosphatase.

### 2.16 miRNAs expression analysis

Total RNA was used to generate cDNA using a Mir‐X miRNA First‐Strand Synthesis RT Kit (Clontech Laboratories, Inc) according to manufacturer's recommendations. The cycle parameters for the RT reaction were 30°C for 10 minutes, 55°C for 5 minutes, 37°C for 1 hour and 85°C for 5 minutes. Subsequently, the synthesized cDNA was used for real‐time quantitative PCR with SensiFAST SYBR Green Kit (Bioline). The reaction was performed using 0.5 μL of template, and the final concentration of primers (Table 2) was 0.4 μmol/L. The following cycling conditions were applied during the reaction: a temperature of 95°C for 10 seconds, followed by 35 cycles at a temperature of 95°C for 5 seconds and annealing temperature of 58.8°C for 20 seconds with a single fluorescence measurement. The mRQ 3′ primer and U6snRNA primers were provided with the RT kit. The average fold change in the gene expression of experimental cultures was compared with control cultures and calculated by the 2 − DDCt method in relation to U6snRNA.

**TABLE 2:** Sequences of primers used for detection of microRNA

Gene	Primer sequence 5′‐3′	Accession no.
*miR‐27a*	AGGGCUUAGCUGCUUGUGAGCA	MIMAT0004633
*miR‐16‐5p*	UAGCAGCACGUAAAUAUUGGCG	MIMAT0000069
*miR‐21‐5p*	UAGCUUAUCAGACUGAUGUUGA	MIMAT0000530
*miR‐29b*	GCUGGUUUCAUAUGGUGGUUUA	MIMAT0004523
*miR‐223*	CGUGUAUUUGACAAGCUGAGUUG	MIMAT0017056
*miR‐320*	GCCUUCUCUUCCCGGUUCUUCC	MIMAT0017057

### 2.17 Statistical analysis

Statistical analysis was performed using GraphPad Prism 5.0. Statistical significance was determined using one‐way analysis of variance (ANOVA) with Dunnett's post hoc multiple comparison test. Asterisk (*) and Hash (#) signs indicated statistical significance in LPS‐induced groups vs healthy control or LPS‐induced control vs *C. glomerata*‐treated groups, respectively. *P* values lower than .05 (*P* < .05) were summarized with one asterisk/hash (*/#), *P* < .01 with two asterisks/hashes (**/##) and *P* < .001 with three asterisks/hashes (***/###).

## 3 RESULTS

### 3.1 Biosorption process of *Mn(II)* ions by algal biomass

The multielemental composition of the solution before and after biosorption of *Mn(II)* ions by *C. glomerata* is presented in Table 3.

**TABLE 3:** The multielemental composition of the algal biomass and the solution before and after biosorption of *Mn(II)* ions (N = 2)

Element and wavelength	Natural *Cladophora glomerata*		Solution before biosorption of *Mn(II)* ions		Solution after biosorption of *Mn(II)* ions	
	Average (mg/g d.m.)	SD	Average (mg/L)	SD	Average (mg/L)	SD
Al 308.215	263	11	0.239	0.057	0.265	0.032
Ca 315.887	148 927	6613	0.840	0.423	48.9	0.1
K 766.491	20 643	1027	0.287	0.064	135	2
Mg 285.213	1749	106	0.120	0.016	7.25	0.21
Mn 257.610	124	5	313	2	247	0.8
Na 588.995	647	1	0.210	0.034	4.20	0.41
P 213.618	947	64	LOD	—	2.32	0.31
S 181.972	13 415	884	181	3	208	7
Si 251.611	636	23	0.122	0.042	0.643	0.058
Zn 213.857	19.2	0.8	0.0826	0.0004	0.0362	0.0070

Abbreviations: d.m., dry biomass; LOD, below low limit of detection; SD, standard deviation.^26^

Biosorption capacity (*q*; mg/g) was evaluated as the difference between the initial concentration (*C*_0_; mg/L) and concentration of metal ions in the solution at time *t*—at equilibrium (*C*_eq_; mg/L) divided per the content of the biomass in the solution (*C*_S_; g/L). For *C. glomerata,* biosorption capacity was equal to 13.2 g of *Mn(II)* ions bound by 1 g of the biomass. Potassium, magnesium, calcium and sodium are the main light metal ions which concentration in the solution after biosorption process significantly increased—by 471, 60, 58 and 20 times, respectively. This indicates that the main mechanism of the biosorption process is the ion exchange between metal ions in the solution (*Mn(II)* ions) and light metal ions naturally bound with functional groups of molecules constituting the algal cell wall.^23^, ^27^

The pH of the solution before biosorption process was 5.035 ± 0.007 and after biosorption process (after sorption equilibrium) 6.537 ± 0.036. This is in agreement with the results presented by Tang et al,^28^ who observed an increase in pH (equilibrium pH higher than the initial pH) for the sorption of metal ions (*Cu(II)*, *Zn(II), Cd(II)* and *Pb(II)*) by the algae—*Oocysis* sp and *Chlorococcum* sp. The increase of pH during the sorption of heavy metals may be explained by the ion‐exchange mechanism according to which there is an exchange between the metal ions originally bound to the functional groups on the algal cell wall (*Na(I), K(I) and Ca(II)*) and weak basic heavy metal ions. This will lead to an increase in pH of the bulk solution from the corresponding shift in the hydrolysis equilibrium of the heavy metal ions.

### 3.2 Extraction of the enriched with *Mn(II)* ions algal biomass

In Table 4, the multielemental composition of the methanolic extract obtained from the enriched with *Mn(II)* ions *C. glomerata* is presented.

**TABLE 4:** The multielemental composition of methanolic extract obtained from the enriched with *Mn(II)* ions algae (N = 2)

Element and wavelength	Methanolic extract	
	Average (mg/L)	SD
Al 308.215	<LOD	<LOD
Ca 315.887	9.82	4.86
K 766.491	28.2	1.0
Mg 285.213	10.9	1.1
Mn 257.610	83.7	2.0
Na 588.995	6.84	1.42
P 213.618	4.99	2.60
S 181.972	33.2	5.0
Zn 213.857	1.04	0.45

### 3.3 Effect of the methanolic extract from *Mn(II)*‐enriched *C. glomerata* on cellular metabolism and morphology in LPS‐induced MC3T3‐E1 Cells

**FIGURE 1:**
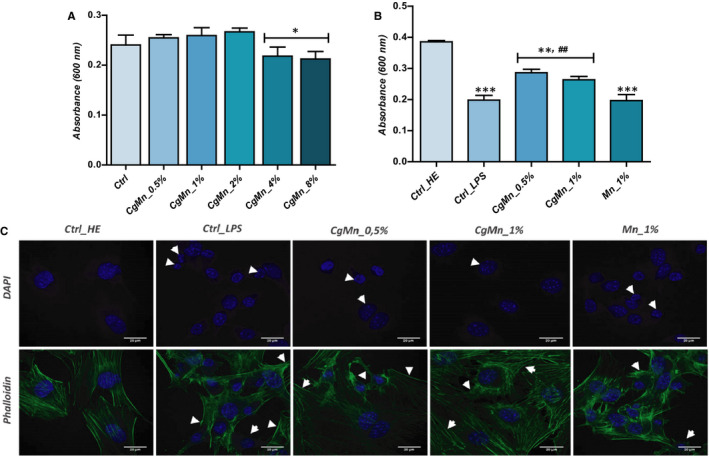
Effect of the methanolic extract obtained from *Mn(II)*‐enriched *Cladophora glomerata* on MC3T3‐E1 viability. A, Histograms represent the average absorbance at 600 nm of the metabolized rezasurin dye after exposure of cells to different concentrations of extract. B, Bar charts represent the mean of the absorbance of the living cells after LPS induction and *C. glomerata* treatment. C, Confocal microscopy imaging of MC3T3‐E1 morphology after each related treatment. DAPI and Phalloidin‐labelled cells were observed using an inverted epi‐fluorescent confocal microscope; scale bar size 20 µm; magnification was set at 60‐fold. The results are expressed as the mean of 3 different experiments ± SD. Asterisk (*) refers to comparison of all treated groups to untreated healthy cells. Hashtag (#) refers to comparison of *C. glomerata* ‐treated groups to LPS‐induced cells. */^#^ *P* < .05, **/^##^ *P* < .01, ***/^###^ *P* < .001

The metabolic activity of MC3T3‐E1 cells cultivated as a mono‐culture in the presence of extract as well as after LPS challenging has been calculated using a Resazurin‐based assay (Figure 1). Cell viability of pre‐osteoblasts was not significantly affected in any of the three 0.5%, 1% and 2% groups; the increase in concentrations up to 4% and 8% of the *Mn(II)*‐enriched *C. glomerata* extract significantly reduced cellular viability rate as compared to the group of untreated cells (*P* < .05), indicating that the extract probably exerts some cytotoxicity toward the tested cells (Figure 1A). Therefore, the two 0.5% and 1% concentrations were selected for the further experiments due to the absence of obvious cytotoxic effects on MC3T3‐E1 cells. *TOX8* assays showed that cell metabolism in MC3T3‐E1 cells exposed to LPS (Figure 1B) was significantly reduced compared to non‐treated culture (*P* < .001). The addition of the two extract concentrations to the challenged cultures minimized the LPS‐induced cytotoxicity (*P* < .01), thereby increasing the level of metabolically active cells in the treated cultures compared to the LPS‐induced group. The evaluation of manganese alone for its part did not exert any improving effect on the condition of MC3T3‐E1 cells stimulated by LPS (Figure 1B).

Confocal images of DAPI‐stained nuclei showed that MC3T3‐E1 cultured in the presence of LPS exhibited reduced cellular confluence, and fragmented nuclei that are characteristic of apoptotic‐undergoing cells (Figure 1C). Moreover, Phalloidin‐labelled F‐actin network clearly demonstrated the appearance of cytoskeleton stress fibres with a general shrinked structure caused by the cytotoxic effects of LPS that were also obvious in clear *Mn(II)* treated cells. Interestingly, the methanolic extract obtained from *Mn(II)*‐enriched *C. glomerata* strongly suppressed the morphological alterations of the pre‐osteoblastic cells induced by LPS. Nuclei were more abundant, rounded with one or more prominent nucleoli; and actin cytoskeleton network was more well defined, dense and homogeneously distributed, while less stress fibres were recorded (Figure 1C).

### 3.4 The methanolic extract prepared from *Mn(II)*‐enriched *C. glomerata* reduces LPS‐induced apoptosis in MC3T3‐E1 pre‐osteoblasts

**FIGURE 2:**
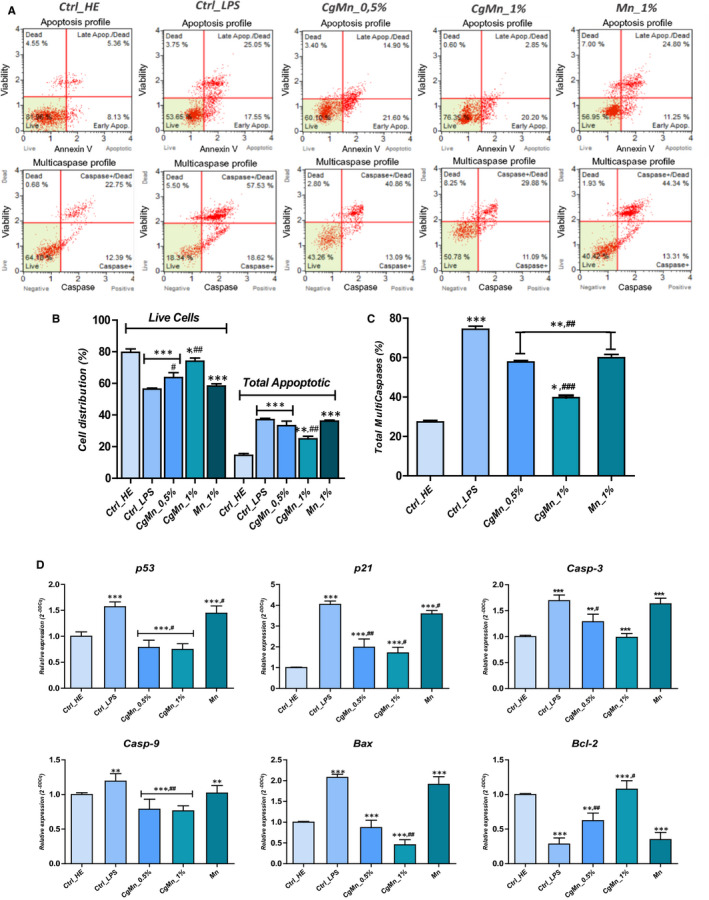
Assessment of apoptosis in LPS‐induced MC3T3‐E1 cells. Cells were assayed with Annexin V/PI staining to measure the percentage of viable cells (Annexin V−/PI−), early apoptotic cells (Annexin V+/PI−), late apoptotic cells (Annexin V+/PI+) and necrotic cells (Annexin V−/PI+). A, Apoptosis and Multicaspases profile plots. Each plot is a representative figure of the three replicates of each determination. B, Bar charts depicting percentage of live and total apoptotic cells (C) Histograms summarizing average of total positive‐activated multicaspases cells. D, Representative bar charts of relative expression of apoptotic key markers. Representative data from three independent experiments are shown ± SD (n = 3). Asterisk (*) refers to comparison of all treated groups to untreated healthy cells. Hashtag (#) refers to comparison of *Cladophora glomerata*‐treated groups to LPS‐induced cells. */^#^ *P* < .05, **/^##^ *P* < .01, ***/^###^ *P* < .001

Following 24 hours of experiment, annexin V staining was detected in MC3T3‐E1 cells treated with 1 µg/mL LPS (Figure 2A) indicating that these cells are undergoing apoptosis. Total live cells (Figure 2B) significantly decreased from 79.61 ± 2.19% in healthy untreated cultures to 56.36 ± 0.66% after LPS challenging (*P* < .001). Moreover, the rate of apoptotic cells increased to 37.10 ± 0.82% in LPS group compared to only 14.50 ± 1.22% in untreated cells (*P* < .001). Treatment of MC3T3‐E1 cultures with the *Mn(II)*‐enriched *C. glomerata* methanolic extract following LPS induction significantly enhanced cellular viability and thus reduced total apoptotic percentage to 24.93 ± 1.63% at a concentration level of 1% (*P* < .01). Clear manganese trace element treatment did not show any significant effect on LPS‐induced apoptosis (Figure 2B).

For further apoptosis assessment, total caspase activity was measured within the cells by flow cytometric‐based assay. Figure 2C showed that, in absence of extract, LPS led to a marked activation of total cellular multicaspases (*P* < .001) of about 74.35 ± 1.58%, which equates to an activation rate of 2.7‐fold in respects to healthy uninduced cells (Figure 2C). However, when the *Mn(II)*‐enriched *C. glomerata* methanolic extract was present, MC3T3‐E1 cells were characterized by strongly reduced multicaspases activity pattern compared with LPS‐control, as evidenced by a lowered activation rate of 1.87‐fold after 1% extract supplementation.

Relative gene expression of key apoptotic factors has been subsequently analysed by RT‐qPCR, and results are represented in Figure 2D. Obtained data were indicated that induction of the apoptosis‐related genes takes place in parallel with the induction of MC3T3‐E1 cells with LPS. All *p53*, *p21*, *Bax*, *Casp‐3* and *Casp‐9* transcripts were significantly up‐regulated following the detrimental effects of LPS. Moreover, a critical down‐regulation of *Bcl‐2* expression was also demonstrated in the same group (*P* < .001). Cultures supplementation with *C. glomerata* methanolic extract containing manganese enhanced the apoptotic status of the LPS‐stressed cells, via the significant lowering of all pro‐apoptotic target genes mRNA levels (*P* < .001), namely *p53*, *p21*, *Casp‐3*, *Casp‐9* and *Bax* (Figure 2D). Furthermore, the antiapoptotic ability of the extract was confirmed by the down‐regulation of the same transcripts in comparison with healthy untreated cells (*P* < .05; *P* < .01). What is more interesting, it appeared that algal extract positively modulated the expression of the *Bcl‐2* survival gene as compared to both LPS and the healthy control groups (*P* < .001; *P* < .05).

### 3.5 Influence of *Mn(II)*‐enriched *C. glomerata* methanolic extract on mitochondrial membrane potential depolarization in LPS‐induced MC3T3‐E1 cells

**FIGURE 3:**
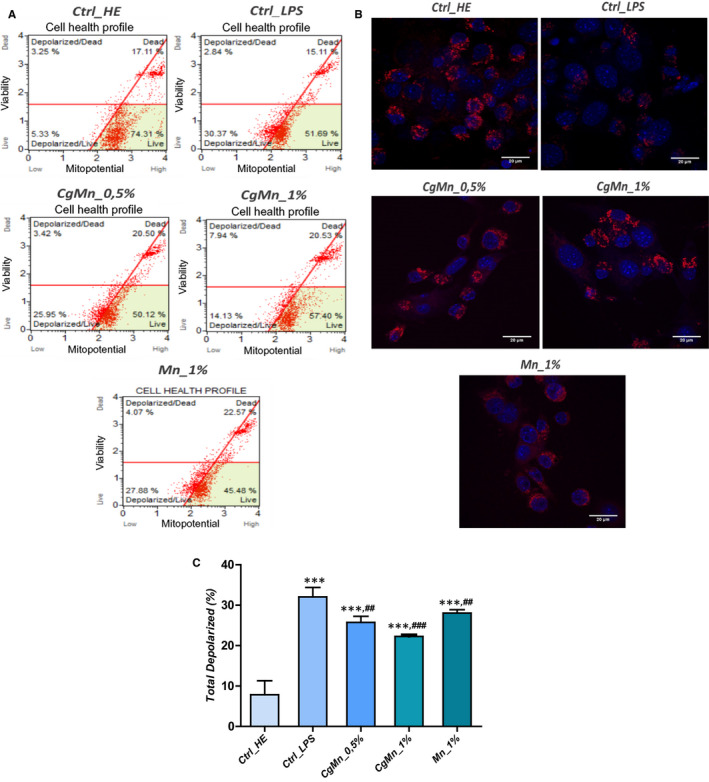
Mitochondrial membrane potential analysis. A, Scattered blots representation of live and dead depolarized cells percentages for one representative experiment. B, Bar charts represent the average percentages ± SD of total depolarization for three repetitions. C, MitoRed stained cells were observed using an inverted epi‐fluorescent confocal microscope; scale bar size 20 µm; magnification was set at 60‐fold. Asterisk (*) refers to comparison of all treated groups to untreated healthy cells. Hashtag (#) refers to comparison of *Cladophora glomerata*‐treated groups to LPS‐induced cells. */^#^ *P* < .05, **/^##^ *P* < .01, ***/^###^ *P* < .001

Mitochondria are known to play a key role to elicit apoptosis in response to many stresses, and the loss of mitochondrial membrane potential represents a hallmark for an early event in apoptosis induction.^29^ In order to investigate whether mitochondrial dysfunctions are involved in LPS‐induced apoptosis, a change of the mitochondrial membrane potential was analysed using a flow cytometric‐based assay. Results in Figure 3B showed a consequent increase in the number of cells with depolarized mitochondrial membranes in LPS‐stressed cells (*P* < .001), the mean percentage raised 32.05 ± 1.31% in challenged group against only 7.85 ± 1.45% monitored in untreated healthy cells, thus suggesting that LPS causes depolarization of mitochondrial potential, which could be linked to the activation of apoptosis in tested cells. A significant positive change in the ΔΨm was observed after *C. glomerata* extract was added to LPS‐induced cultures. Evident MMP restauration was demonstrated by a decrease in the rate of depolarized mitochondrial cells up to 22.27 ± 0.43% for 1% methanolic‐treated MC3T3‐E1 cells. The concentration of 0.5% extract proved to be less effective; however, the rate of depolarized‐positive cells appeared to be lower than that of the untreated cells. Confocal imaging of metabolically active mitochondria confirmed the above results, where a larger number of MitoRed‐labelled red fluorescent spots were detected in the cytoplasm of the cells exposed to algal extract, as opposed to the LPS‐induced cells, which were characterized by a significant reduction in the fluorescence intensity emitted by the dye (Figure 3C).

### 3.6 Effect of the methanolic extract obtained from *Mn(II)*‐enriched *C. glomerata* on cellular oxidative stress

Involvement of reactive oxygen species (ROS) in the LPS‐induced MC3T3‐E1 cytotoxicity was evaluated using the flow cytometric‐based fluorescent staining.

**FIGURE 4:**
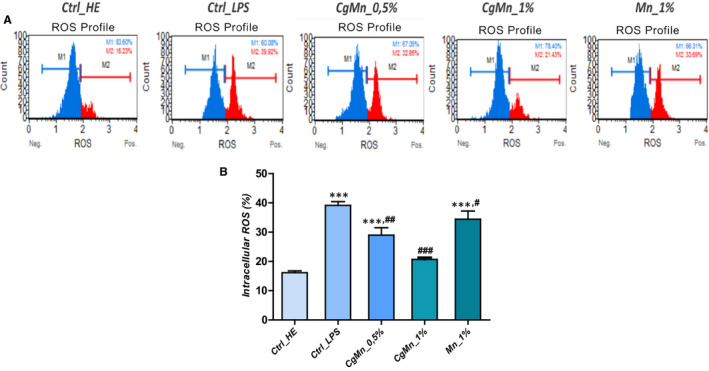
*Cladophora glomerata* reduces oxidative stress during LPS challenging of MC3T3‐E1. A, Representative plots depicting cell stained with DHE evaluated using a flow cytometer. B, Each bar summarizes the mean percentage ± SD of three independent experiments for total intracellular ROS‐positive cells. Asterisk (*) refers to comparison of all treated groups to untreated healthy cells. Hashtag (#) refers to comparison of *C. glomerata* ‐treated groups to LPS‐induced cells. */^#^ *P* < .05, **/^##^ *P* < .01, ***/^###^ *P* < .001

The basal levels of fluorescence of the DCF dye were significantly increased up to 41 ± 1.01% after LPS treatment of MC3T3‐E1 cultures (Figure 4B) as compared to uninduced control cells (*P* < .001). The methanolic extract obtained from *Mn(II)*‐enriched *C. glomerata* exhibited strong antioxidant effect; indeed, at the 1% concentration of extract, the rate of ROS‐positive cells appeared comparable to that of the healthy control cells, while being statistically different from that of the LPS‐treated cells (*P* < .001). Less significant effect was recorded for the group exposed to 0.5% extract, where the number of positive fluorescent cells appeared to be higher than that of the normal cells (*P* < .001), but still remained less than that of the stressed cells (*P* < .01). Concerning the group that received the clear *Mn(II)* ions, similar trend to that of the 0.5% extract group was observed (Figure 4B).

### 3.7 Effect of *Mn(II)*‐enriched *C glomerate* methanolic extract on the mRNA expression of osteoblast‐specific genes in LPS‐induced MC3T3‐E1 cells

**FIGURE 5:**
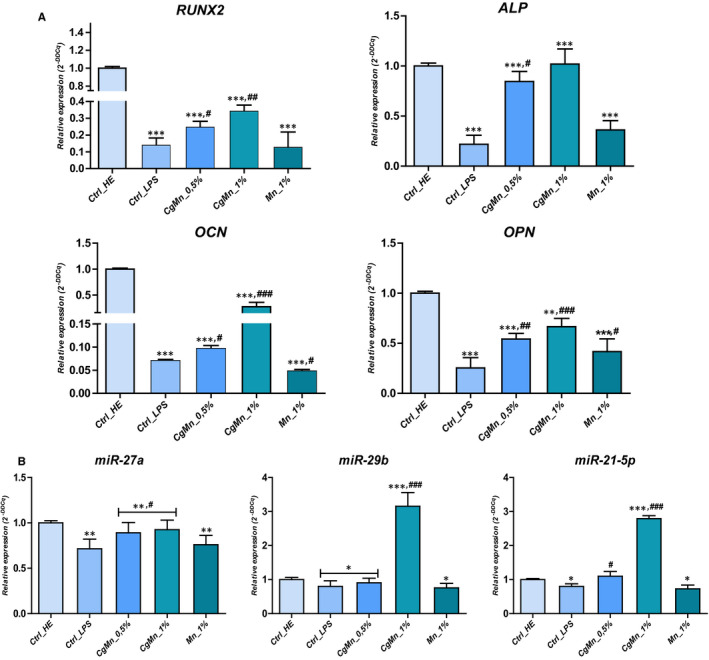
Relative mRNA expression analysis of the osteogenic‐specific marker genes (A) RUNX2, ALPS, OCN and OPN, (B) miR‐27a, miR‐29b and miR‐21‐5p in the *Cladophora glomerata*‐treated and untreated LPS‐induced MC3T3‐E1. The results are expressed as the mean of 3 different experiments ± SD. Asterisk (*) refers to comparison of all treated groups to untreated healthy cells. Hashtag (#) refers to comparison of *C. glomerata*‐treated groups to LPS‐induced cells. */^#^ *P* < .05, **/^##^ *P* < .01, ***/^###^ *P* < .001

To determine the effect of algal extract enriched with the trace element *Mn(II)* on LPS‐induced MC3T3‐E1 cells, detection of mRNA levels of key osteogenic markers, including *ALP*, *OCN*, *OPN* and *RUNX2*, was performed through real‐time PCR. LPS obviously decreased osteogenic‐related factors expression (*P* < .001), as indicated by the significantly reduced levels of *Runx2*, *OCN*, *OPN* and *ALP* transcripts (Figure 5A).

The obtained data showed that treatment with tested extract alleviated LPS‐reduced osteogenic potential of MC3T3‐E1, as evidenced by the significant up‐regulation of mRNA expression (*P* < .001) of the osteoblast‐specific genes namely, *ALP*, *Runx2*, *OPN* and *OCN* in a dose‐dependent manner after 24 hours incubation, as compared to the non‐*C. glomerata* extract‐treated group challenged with LPS. These results suggested that *C. glomerata* after its biomass enrichment with *Mn(II)* ions may promote the early stage of osteoblast differentiation in MC3T3‐E1 cells. The profiling of different miRNA expression panel that is involved in osteogenesis regulation was also established by means of RT‐PCR (Figure 5B). As expected, LPS significantly decreased the expression levels of both *miR‐27a*, *miR‐29b* and *miR‐21‐5p* in opposition to untreated healthy cultures (*P* < .05). The supplementation of cultures with freshwater algae extract has positively modulated the transcription of these same microRNAs in a dose‐dependent manner; however, the most remarkable effect was observed at cultures treated with 1% extract, where the expression of *miR‐27a*, *miR‐29b* and *miR‐21‐5p* was up‐regulated by approximately 1.3‐fold, 3.9‐fold and 3.48‐fold, respectively, in comparison with the group induced by LPS (Figure 5B).

### 3.8 Effect of the methanolic extract obtained from *Mn(II)*‐enriched *C. glomerata* on the mRNA expression of osteoclast‐related genes in LPS‐induced MC3T3‐E1 cells

**FIGURE 6:**
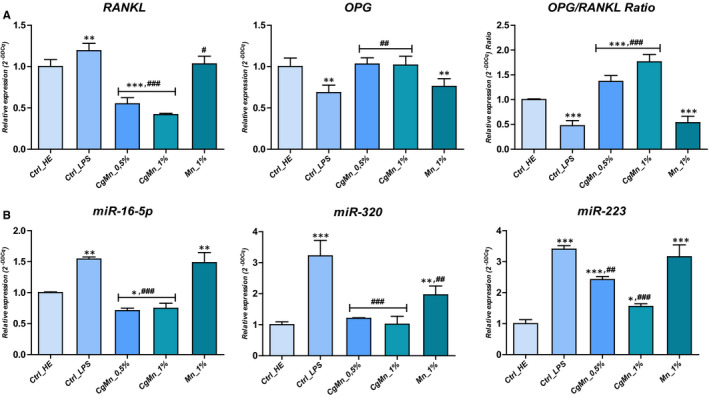
Relative mRNA expression analysis of the osteoclastogenic‐specific marker genes RANKL, OPG, miR‐16‐5p, miR‐223 and miR‐320, and OPG/RANKL ratio in the *Cladophora glomerata*‐treated and untreated LPS‐induced MC3T3‐E1. The results are expressed as the mean of 3 different experiments ± SD. Asterisk (*) refers to comparison of all treated groups to untreated healthy cells. Hashtag (#) refers to comparison of *C. glomerata*‐treated groups to LPS‐induced cells. */^#^ *P* < .05, **/^##^ *P* < .01, ***/^###^ *P* < .001

To further elucidate the role of *C. glomerata* extract in LPS‐induced MC3T3‐E1 osteoclastogenic pathways modulation, the expressions of osteoclastic markers genes in pre‐osteoblastogenic cells with or without algal extract coculture were detected (Figure 6A). Results showed that expression of *RANKL* was significantly up‐regulated upon exposure of cells to LPS (*P* < .001). The mRNA level of the same transcript markedly decreased after the methanolic extract from *Mn(II)*‐enriched *C. glomerata* was added, in a dose‐dependent manner (*P* < .001), 1% concentration being the most effective in regulation of pro‐osteoclastogenic marker expression. Transcription rate of the *RANKL* antagonist *OPG* factor was significantly modulated in the LPS‐challenged group, as compared to healthy cells (*P* < .001). Treatments with *Mn(II)*‐enriched *C glomerate* methanolic extract successfully restored the basal expression of *OPG*, which appeared to be comparable to that in normal untreated control group. Expression ratio of *OPG* to *RANKL* (Figure 6A) demonstrated similar changes levels due to decreased *OPG* expression level and an elevated *RANKL* expression level, in osteoblasts in response to treatment with LPS. Subsequent *Mn(II)*‐enriched *C. glomerate* methanolic extract application resulted in statistical significant increase in *OPG/RANKL* ratio when compared to both LPS‐treated and untreated MC3T3‐E1 cells (*P* < .001).

Similar trends of overexpression in osteoclastogenesis inducing microRNAs (*P* < .001), namely *miR‐16‐5p*, *miR‐320* and *miR‐223* were observed after analysis of LPS‐stressed cells (Figure 6B). Application of *Cladophora* extract made it possible to sensibly regulate the transcription of the osteoclastogenic‐related microRNAs, suggesting that this extract may attenuate the signalling pathways that initiate aberrant osteoclastogenic differentiation.

### 3.9 Effect of methanolic extract of *Mn(II)*‐enriched *C. glomerata* on LPS‐induced MC3T3‐E1_4B12 coculture osteoclastogenic differentiation

**FIGURE 7:**
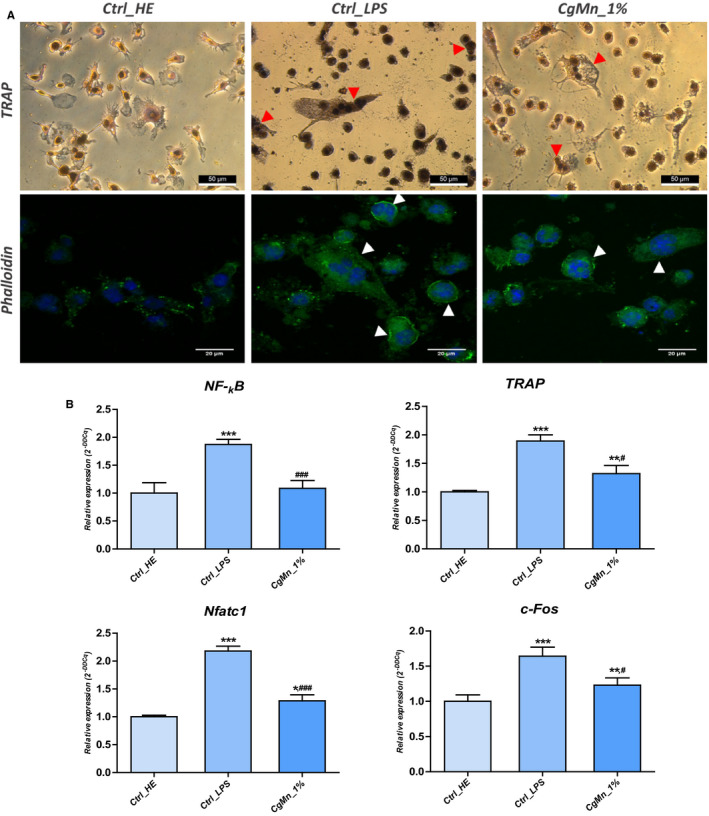
Evaluation of osteoclastogenesis in 4B12 cells. A, 4B12 cultures were stained with TRAP staining and observed under an inverted microscope, bar size 50 µm; magnification was set at 40‐fold. Phalloidin‐labelled cells were observed using an inverted epi‐fluorescent confocal microscope; scale bar size 20 µm; magnification was set at 60‐fold. B, Representative bar charts of relative expression of osteoclastogenic‐related genes. Representative data from three independent experiments are shown ± SD (n = 3). Asterisk (*) refers to comparison of all treated groups to untreated healthy cells. Hashtag (#) refers to comparison of *Cladophora glomerata*‐treated groups to LPS‐induced cells. */^#^ *P* < .05, **/^##^ *P* < .01, ***/^###^ *P* < .001

To study the ability of LPS‐induced osteoblasts to support osteoclast formation, MC3T3‐E1 cells were induced by LPS to stimulate the production of pro‐osteoclastogenic factors and cocultured with 4B12 pre‐osteoclastogenic cells in order to induce their differentiation. LPS challenging of MC3T3‐E1 strongly enhanced 4B12 osteoclastogenic differentiation as evidenced by the increased stimulation of TRAP‐positive mononuclear, as well as multinucleated osteoclastic cells, within the coculture system as compared to the untreated cultures (Figure 7A). In order to demonstrate whether the *C. glomerata* extract could counter osteoclastogenic differentiation, the established coculture system was supplemented with the most effective concentration that was 1%. Photomicrographs clearly showed reduced number of TRAP‐positive cells with lower corresponding TRAP amber staining intensity, suggesting that present extract suppressed LPS‐induced differentiation of 4B12 cells into osteoclasts. Confocal imaging of Phalloidin‐labelled osteoclastogenic cultures evidenced a characteristic rearrangement of the F‐actin network to form well‐defined rings in LPS‐induced coculture systems (Figure 7A), whereas, cultures treated with algal extract displayed mainly disrupted actin rings. Real‐time RT‐PCR experiments further demonstrated that there were about 1.87‐fold, 1.72‐fold, 2.18‐fold and 1.38‐fold increase in the expression pattern of main osteoclastogenic factors *NF‐κB*, *TRAP*, Nuclear Factor Of Activated T Cells 1 and *c‐Foc*, respectively, after LPS elicitation relative to healthy uninduced cells (Figure 7B). 1% of the methanolic extract from *Mn(II)*‐enriched *C. glomerata* exerted a consistent suppressive effect (*P* < .05, *P* < .01, *P* < .001) on the expression of osteoclastogenic differentiation regulating factors mRNAs that were stimulated subsequently to the production of initiating molecules such as *RANKL* by MC3T3‐E1 cells under the influence of LPS (Figure 7B).

## 4 DISCUSSION

Excessive bone resorption during chronic inflammatory pathologies, such as septic arthritis and osteomyelitis, is usually initiated following activation of bacterial‐induced inflammatory responses.^30^ LPS, a pro‐inflammatory glycolipid component of the gram‐negative bacterial cell wall, is a mediator widely implicated in the gram‐negative bacterial bone destruction and alteration process.^31^ In the present study, we demonstrated that *Mn(II)*‐enriched *C. glomerata* methanolic extract can alleviate LPS‐induced osteoblastic dysfunctions in MC3T3‐E1 cells. In addition, algal extract attenuated LPS‐induced MC3T3‐E1‐mediated 4B12 osteoclastogenic differentiation. It has been well documented that exposure to LPS triggers to inhibition of osteoblast differentiation and causes different cellular dysfunctions.^32^ Presented data indicated that culture of MC3T3‐E1 in the presence of LPS strongly affected the cell viability as evidenced by reduced resazurin metabolization, and directly induced apoptosis, loss of MMP and excessive ROS production.

Induction of osteoblast apoptosis following bacterial infection irreversibly leads to pathological bone destruction. Increased of total apoptotic population after LPS treatment was accompanied by significantly up‐regulated mRNA expression levels of pro‐apoptotic factors namely *p53*, *p21*, *Bax*, *caspase‐3* and *caspase‐9*, whereas *Bcl‐2* expression appeared to be highly decreased in MC3T3‐E1 cells; moreover, LPS application resulted in the activation of different caspase complexes that are closely involved in apoptosis. Our data showed that treatment with *Mn(II)*‐enriched *C. glomerata* extract inhibited LPS‐induced osteoblast apoptosis, and significantly restored the down‐regulated expression of *Bcl‐2* while decreasing the up‐regulated expression of *p53*, *p21*, *Bax*, *caspase‐3* and *caspase‐9*, compared to non‐extract‐treated cultures of MC3T3‐E1 cells induced by LPS. These results indicated that supplementation with *Mn(II)*‐enriched *C. glomerata* extract could have a protective effect against LPS‐induced osteoblast apoptosis. Previous studies have indeed already highlighted the strong anti‐apoptotic effect of the macroalgae in different cellular models including equine ASCs cells affected with metabolic syndrome that are known to be prone to significant apoptosis.^19^, ^25^ Recent studies have elucidated that normal mitochondrial function is importantly required for osteogenic differentiation, and its dysfunction has already been associated with impaired osteogenesis.^33^, ^34^, ^35^ The toxic effect produced by LPS was demonstrated to be closely associated with mitochondrial dysfunction.^36^ In this investigation, LPS induced dramatical MMP collapse in MC3T3‐E1, as well as reduced mitochondrial activity as evidenced trough decreased fluorescent signal emitted by MitoRed dye. Mitochondrial integrity responds to control network exerted by a cluster of proteins belonging to the *Bcl‐2* family. Members of this family, such as *Bcl‐2* and *Bcl‐XL*, possess four conserved *Bcl‐2* homology (BH) domains, designated BH1 through BH4. It is widely accepted that the BH4 domain protects mitochondria from apoptotic signals.^37^ Collapse of the *Bcl‐2* gene expression following LPS application could trigger to the loss of mitochondrial integrity and membrane depolarization, and thus to an initiation of apoptosis via the release of apoptogenic factors such as cytochrome c into the cytoplasm. Subsequently, cytochrome c, caspase‐9 and apoptosis protease activating factor‐1 (*Apaf‐1*) together form the apoptosome, which processes after that to the executioner caspase‐3.^38^ After exposure to *C. glomerata* extract, we found obvious increased mitochondrial membrane potentiation, as well as high MitoRed fluorescent signal corresponding to increased number of active mitochondria. These results indicate that algal extract caused a significant improvement of cellular mitochondria function, which is in accordance with our previous finding.^19^, ^25^ One of the supposed mechanisms by which the algae extract could exert effect on the maintenance of the mitochondrial integrity, lies in the potential of the latter to modulate positively and to potentiate the transcription of the *Bcl‐2* cell survival gene, which would restore the protective effect of that factor on mitochondria.

Recent reports have suggested that LPS stimulates the intracellular ROS production within the cells through the activation of NADPH oxidase. These free radicals seem to subsequently serve as second messenger to enhance LPS‐induced cellular dysfunctions.^39^ ROS, including superoxide anion, hydroxyl radical and their by‐products as well, may play dual roles in the course of osteomyelitis. While intended to attack invading pathogens, ROS generated within mitochondria in excess are also toxic to cells such as osteoblasts through the initiation of lipid peroxidation, DNA fragmentation and protein oxidation.^40^ On the other hand, ROS can activate diverse downstream signalling molecules such as protein kinase C, mitogen‐activated protein kinase (*MAPK*) and nuclear factor‐κB (*NF‐κB*) to regulate the expression of genes encoding a variety of pro‐inflammatory and osteoclastogenic factors.^41^ In the present study, MC3T3‐E1 cells challenged with LPS, where markedly prone to intracellular ROS overproduction, as a consequence of endotoxin toxicity. Relative treatment of induced cultures with *Mn(II)*‐enriched algal extract exerted a significant antioxidant effect, by reducing the levels of ROS‐positive cells. When mitochondria are impaired or dysfunctional, ROS production is inevitably further increased what subsequently exacerbate the oxidative stress in mitochondria.^42^ Manganese is a trace element specially required for MnSOD enzyme in order to reduce mitochondrial oxidative stress. Additionally, MnSOD is a primary antioxidant that scavenges superoxide formed within the mitochondria and protects against oxidative stress. In this prospect, it can be assumed that the biosorption process that was performed on the *C. glomerata* biomass, was effective in enhancing the biological properties of the algae, particularly in the improvement of the antioxidant status of the extract as well as the cells themselves.^15^, ^43^

The inhibitory effect of LPS on osteoblast differentiation pathway was investigated by evaluating mRNA expression levels of osteoblast marker genes. Transcripts levels of *ALP*, *OCN*, *OPN*, *Runx2*, as well as *miR‐27a*, *miR‐29b* and *miR‐21* in MC3T3‐E1 cells were significantly down‐regulated by LPS. Previous investigations already reported on the well‐established pattern of osteogenic markers which generally includes *ALP*, *OCN*, *OPN* and *Runx2*, all of which are closely related to bone formation and are widely used for inducing osteogenic differentiation.^44^ The osteogenesis signalling regulatory networks include also various other components, among of them, micro (mi)RNAs, that form feedback loops controlling the balance of osteogenic differentiation by positive or negative regulation.^45^ *ALP* and *Runx2* have been suggested to be involved in the early‐stage molecular events of osteoblast differentiation, whereas *OCN* and *OPN* were involved in the late‐stage molecular events; moreover, targeted miroRNAs have been showed to activate and regulate the *Wnt* signalling pathway, which promotes osteoblast differentiation. *miR‐27a* is known to target *APC*, which leads to accumulation of β‐catenin and activation of *Wnt* signalling for osteoblast differentiation promotion.^46^ *miR‐29b* participates in the fine‐tuning of *Wnt* signalling such as canonical Wnt signalling and down‐regulates in the same time inhibitors of canonical Wnt signalling such as *DKK1*, *Kremen2* and *SFRP2* to promote osteoblast differentiation.^47^ Transcription of *miR‐21* results in bone formation mainly through the modulation of *Smad7‐Smad1/5/8‐Runx2* pathway.^48^ The protective effect of treatment with *Mn(II)*‐enriched *C. glomerata* extract on the LPS‐induced dysfunctional osteoblast differentiation pathway was characterized by the reversal of the LPS‐detrimental effect on the transcription of the osteogenesis key genes; thus, tested extract strongly promoted the expression of both mRNA and microRNAs that are essential to MC3T3‐E1 differentiation. Manganese plays an essential role as a co‐factor in the formation of bone cartilage and bone collagen, as well as in bone mineralization. Various studies have demonstrated that lack of manganese is responsible in cartilage formation disturbances and produce osteopenia, as result of an imbalance between osteoblastic and osteoclastic activity.^49^ Furthermore, as *Mn(II)* ions mainly activate phosphatases, kinases, decarboxylases and glycosyltransferases in different tissues such as bone, it is believed that skeleton abnormalities could be partly due to a reduction in proteoglycan synthesis secondary to a reduction of manganese dependent glycosyltransferases.^50^ The positive regulatory effect of tested algal extract on expression patterns of main pro‐osteogenic factors might be favoured by the combination of algae's secondary metabolites such as polyphenols and fatty acids and manganese fixed by biosorption.

During bone formation, inclining the balance beneficial to the osteoclasts differentiation usually results in excessive bone absorption and triggers to osteoclasts‐related skeleton abnormalities.^51^ Osteoclasts are basically monocyte‐macrophage lineage‐derived that are initiated following the interactions between osteoblasts/bone marrow stromal cells and osteoclast precursors. Two molecules that are mainly produced by osteoblasts/marrow stromal cells are essential and sufficient to support osteoclastogenesis: macrophage colony‐stimulating factor (*M‐CSF*) and *RANKL*. Meanwhile, osteoprotegerin (OPG), which is a soluble factor secreted by osteoblasts and osteoblast‐like cells, acts as a receptor antagonist for *RANKL*, in order to prevent it binding to *RANK* and thus decreases osteoclastogenesis.^52^

During the present investigation, LPS application was found to highly stimulate the production of the pro‐osteoclastogenic factor *RANKL*, as well as *miR‐16‐5p*, *miR‐223* and *miR‐320* microRNAs, and to strongly impair *OPG* gene expression in MC3T3‐E1 pre‐osteoblasts. The addition of *C. glomerata* extract potently regulated the expression of the above genes, exhibiting thus a remarkable anti‐osteoclastogenic potential on MC3T3‐E1 cells. Binding of *RANKL* to activator of nuclear factor κB (*RANK*) receptor, subsequently, activates transcription factors including *c‐Fos* and nuclear factor of activated T‐cell cytoplasmic 1 (Nuclear Factor Of Activated T Cells 1) that are known to be essential to osteoclasts formation.^53^ These factors are able to regulate and induce several osteoclastogenesis‐related genes such as tartrate‐resistant acid phosphatase (*TRAP*), cathepsin K (*CTK*), calcitonin receptor (*CTR*) and matrix metallopeptidase‐9 (*MMP‐9*).^54^ In another hand, the implication of microRNAs in osteogenesis impairment has also been pointed out; up‐regulation of *miR‐16* leads to the blocking of the *Wnt* signal pathway through *Wnt5a* repression.^55^, ^56^ High levels of *miR‐223* decrease the expression of *Runx2*, *OPN* and *OCN* mediators that are necessary for osteoblasts differentiation.^57^ *miR‐320* has also been extensively studied in osteoblastic cell function. This miRNA may impair the osteoblast differentiation by targeting key bone‐formation genes such as *CTNNB1* (B‐catenin) and *Runx2*; moreover, its overexpression is closely related to increased stress oxidation.^58^

The established coculture system of the present study aimed to simulate the signalling cascades leading to osteoclastogenesis in the course of osteomyelitis. Induction of MC3T3‐E1 cells with LPS, which caused the release of pro‐osteoclastogenic factors, strongly activated the formation of osteoclasts on precursor 4B12 cells, as highlighted by the emergence of positive‐TRAP mononucleated and multinucleated osteoclasts in coculture system. From the many genes and factors that positively regulate and promote osteoclastogenesis and osteoclast activation, *Mn(II)*‐enriched *C. glomerata* methanolic extract was able to significantly down‐regulate the expression of the osteoclastogenic‐specific studied genes panel, which comprises both *NF‐κB*, *TRAP*, Nuclear Factor Of Activated T Cells 1 and *c‐Fos*, and thereby interfere in the process of osteoclastogenesis. *NF‐κB* has been implicated in the mediation of *RANKL*‐induced osteoclast differentiation, leading to activation of *Fos* proto‐oncogene (*c‐Fos*) and Nuclear Factor Of Activated T Cells 1, which is considered as the master regulator of osteoclast formation, that regulates a number of osteoclast specific genes such as *TRAP*, cathepsin K, calcitonin receptor and osteoclast‐associated receptor (*OSCAR*).^59^ *TRAP*, which is a di‐iron‐containing metalloenzyme largely expressed in osteoclasts, is subsequently found to be dramatically up‐transcribed during osteoclast differentiation following Nuclear Factor Of Activated T Cells 1 recruitment to the *TRAP* promoter.^60^ Although the present research has been able to demonstrate a promising effect of the *C. glomerata* extract enriched with manganese in the regulation of osteogenesis, as well as modulation of osteoclastogenesis, the implementation of additional tests, including analysis of mineralization using both Alizarin red and von Kossa‐based assays, as well as osteogenic and osteoclastogenic‐related proteins profiling by means of Western blot technique, would provide more interesting data on the extract‐associated underlying molecular mechanisms of action and thus open new perspectives in the development of new therapeutic agents for the treatment of osteomyelitis.

## 5 CONCLUSION

The present investigation showed the significant effectiveness of *C. glomerata* methanolic extract prepared from biomass, which was previously enriched by biosorption in *Mn(II)* ions in the protection against LPS‐induced osteoblast dysfunctions mainly characterized by prominent apoptosis, loss of MMP, excess in ROS production and collapse in gene expression of main osteogenic‐specific factors. Moreover, extract was successful in preventing osteoclasts formation and activation, on LPS‐triggered osteoclastogenesis of MC3T3‐E—4B12 coculture system. These results indicate that use of *C. glomerata* as well as trace elements biosorption technology may be a potential alternative for the prevention of abnormal bone loss induced by LPS in chronic inflammatory diseases and osteomyelitis.

## CONFLICT OF INTERESTS

Not applicable.

## AUTHOR CONTRIBUTIONS

LB and KM designed the research and validated results. LB, MB and KK performed experiments, LB collected and analysed data. IM performed natural extract preparation and analysis. LB and IM wrote manuscript text and prepared figures and tables. KM and IM have read critically and edited the manuscript. KM acquired funding. All authors reviewed and approved final version of manuscript.

## ETHICS APPROVAL AND CONSENT TO PARTICIPATE

Not applicable.

## CONSENT FOR PUBLICATION

Not applicable.

## Data Availability

All data sets generated and/or analysed during the current study are presented in the article, the accompanying Source Data or Supplementary Information files, or are available from the corresponding author upon reasonable request.
